# Profiles of immune infiltration in colorectal cancer and their clinical significant: A gene expression‐based study

**DOI:** 10.1002/cam4.1745

**Published:** 2018-08-16

**Authors:** Yongfu Xiong, Kang Wang, He Zhou, Linglong Peng, Wenxian You, Zhongxue Fu

**Affiliations:** ^1^ Department of Gastrointestinal Surgery The First Affiliated Hospital of Chongqing Medical University Chongqing China; ^2^ Department of Breast Surgery The First Affiliated Hospital of Chongqing Medical University Chongqing China

**Keywords:** clinicopathological features, colorectal cancer, genomic signature, nomogram

## Abstract

Immune infiltration of colorectal cancer (CRC) is closely associated with clinical outcome. However, previous work has not accounted for the diversity of functionally distinct cell types that make up the immune response. In this study, based on a deconvolution algorithm (known as CIBERSORT) and clinical annotated expression profiles, we comprehensively analyzed the tumor‐infiltrating immune cells present in CRC for the first time. The fraction of 22 immune cells subpopulations was evaluated to determine the associations between each cell type and survival and response to chemotherapy. As a result, profiles of immune infiltration vary significantly between paired cancer and paracancerous tissue and the variation could characterize the individual differences. Of the cell subpopulations investigated, tumors lacking M1 macrophages or with an increased number of M2 macrophages, eosinophils, and neutrophils were associated with the poor prognosis. Unsupervised clustering analysis using immune cell proportions revealed five subgroups of tumors, largely defined by the balance between macrophages M1, M2, and NK resting cells, with distinct survival patterns, and associated with well‐established molecular subtype. Collectively, our data suggest that subtle differences in the cellular composition of the immune infiltrate in CRC appear to exist, and these differences are likely to be important determinants of both prognosis and response to treatment.

## 1 INTRODUCTION

Despite advances in screening, diagnosis, and curative resection, colorectal cancer (CRC) is still one of the leading causes of cancer‐related death worldwide, and its clinical outcome for individual cases remains unsatisfactory.^1^ At present, surgical resection is the only potentially curative therapy for CRC. Unfortunately, about 20‐25% of patients with newly diagnosed CRC present with distant metastases, and only a small population of these patients can undergo curative operation.^2^ In addition, approximately 50% of CRC patients with resectable tumors will develop recurrence, most within 2 years.^3^ As a well‐recognized heterogeneous disease, the phenotype and prognostic diversity of CRC present great challenges in making individualized clinical decision.^4^ During the past decades, genomic and biological changes in cancer cells have been extensively investigated to identify patient subgroups with different prognosis and treatment response, as well as to find potential drug targets.^5^, ^6^ However, the malignant phenotypes of cancers are defined not only by the intrinsic activities of tumor cells but also by the immune cells recruited to and activated in the tumor‐related microenvironment.^7^ Until recently, the roles of immune cells in the tumor‐related microenvironment remain poorly understood.

Tumor‐infiltrating immune cells (TIICs), whose function and composition subtly altered according to the immune status of the host, have been reported to be effectively targeted by drugs and to correlate with the clinical outcome.^2^, ^8^, ^9^ Mechanism studies confirmed that between TIICs and malignant cells in the tumor stroma, there is in fact a complex interaction which has significant prognostic relevance as the immune system has a dual role by supporting both host defense and tumor progression.^10^ As an immunosensitive tumor, CRC infiltrated by a heterogeneous collection of TIICs, including T cells, dendritic cells, macrophages, neutrophils, and mast cells, and it has been frequently reported that the type, density, and location of TIICs within CRC present with great prognostic value.^11^, ^12^, ^13^ For example, Klintrup et al^14^ evaluated the overall inflammatory cell reaction and the density of each TIICs type in a large cohort of CRC patients. Their immunohistochemistry experiment revealed that inflammatory reaction at the invasive margin was generally the most significant predictor for both overall survival (OS) and disease‐free survival (DFS).^14^ Additionally, mature T cells and dendritic cells, memory T cells were commonly detected TIICs subpopulation with favorable prognostic, while immune suppressive regulatory T cells are associated with impaired prognosis in CRC.^15^, ^16^, ^17^ More importantly, several recent studies even pointed out that immunological data (the type, density, and location of immune cells within the tumor samples) are a better predictor of patient survival than the histopathological methods currently used to stage colorectal cancer.^17^, ^18^

However, we noted that the prognostic value of certain TIICs subpopulation does not always consistent with each other even in studies with same experiment design. For instance, one study provided evidence for a role of FoxP3^+^ Treg density in CRC tissue as predictor of prolonged survival.^19^ While in another study, the expression of Tregs surface markers cluster (FoxP3, TGF‐b, IL‐10) was not associated with a particular outcome.^20^ These results were validated by genomic and in situ immunohistochemistry analyses. In addition, IL‐17 has been shown to have a dual role in cancer by promoting and inhibiting tumor growth, whereas in various human cancers, it has been described both as “good” and “bad” prognostic factor. Thus, the function and prognostic value of IL‐17^+^ T cells were also mutually contradictory in different studies.^17^

Potential explanations for above contradictory results are technical limitations. The immune response is characterized by numerous specialized cell types that interact in a highly coordinated manner. However, previous studies have been limited to a very narrow view of immune response. They evaluated TIICs by immunohistochemistry‐based analysis, which depends on a single marker to identify one specific TIICs subpopulation. Obviously, this approach can be misleading and are not comprehensive as many marker proteins are expressed in different cell types. More importantly, immunohistochemistry is considerably less effective for detecting cell types with quite a small number and discriminating closely related cell types. Thus, no previous study has shed light on the prognostic value of these TIICs subpopulation.

In recent, a metagene approach, known as CIBERSORT, was developed.^21^ This gene expression‐based deconvolution algorithm assesses the relative expression changes of a set of genes compared with the expression of all other genes in a sample. Therefore, the diversity and the landscape of TIICs can be properly determined by this method. Due to the superior performance of CIBERSORT, its application in studying cell heterogeneity has aroused increasing attention.^22^, ^23^, ^24^ In this study, we therefore applied CIBERSORT, for the first time, to quantify the 22 TIICs subsets of immune response in CRC in order to investigate its relationship with molecular subpopulation, survival, and response to chemotherapy. It is hoped that this research will offer some important insights into the complex relationship between the heterogeneity of intratumoral immune cells, tumor molecular subtypes, and disease progression in CRC.

## 2 MATERIALS AND METHODS

### 2.1 Data acquisition

**Figure 1:**
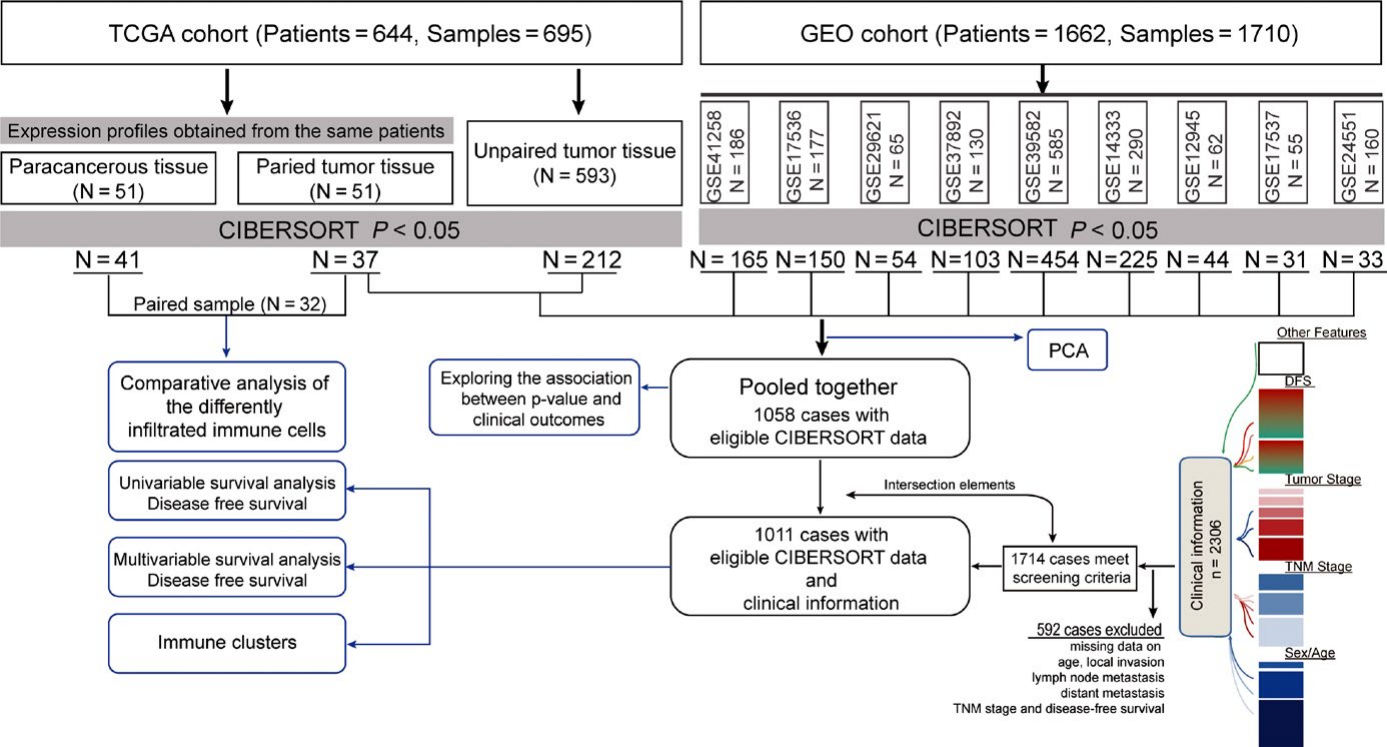
Flowchart detailing the study design and samples at each stage of analysis. TGCA, The Cancer Genome Atlas; GEO, Gene Expression Omnibus; CIBERSORT, Cell type Identification By Estimating Relative Subsets Of known RNA Transcripts; DFS, disease‐free survival

This study made use of data in the public domain. Publicly available datasets with gene expression profiles and corresponding prognosis information from CRC patient‐derived tumor and normal tissues were identified and downloaded from GEO^25^ and TCGA,^26^ uploaded up to 31 December 2017. Datasets with small sample size (N < 50) and duplications were excluded. For some datasets whose prognosis information were not with their expression profiles, we either searched the supplements or contacted one or more of the investigators to get the missing information. Subsequently, expression profiles of each sample and corresponding clinical data were manually organized. Only patients diagnosed with colorectal cancer, and with clinicopathological and survival information available, were included. In addition, patients with any missing or insufficient data on age, local invasion, lymph node metastasis, distant metastasis, TNM stage, and disease‐free survival were also excluded from subsequent processing. Preprocessing and aggregation of raw data were performed according to the robust multi‐array average algorithm. For the TCGA dataset, RNA sequencing data were firstly transformed using voom^27^ (variance modeling at the observational level), converting count data to values more similar to those resulting from microarrays. As we pooled numerous studies published over some years, the technologies used to measure gene expression differed substantially. Thus, analysis in the present study was confined to samples measured via the Affymetrix HG‐U133 or Illumina Hiseq platforms. Quality control of the resulting expression data was executed as previously described.^28^, ^29^ Our study followed the Reporting Recommendations for Tumor Marker Prognostic Studies (REMARK) criteria as listed in their guidelines.^30^ Details of the study design and which samples were included at each stage of analysis are illustrated in Figure 1 as a flowchart.

### 2.2 Evaluation of tumor‐infiltrating immune cells

Normalized gene expression data were used to infer the relative proportions of 22 types of infiltrating immune cells using the CIBERSORT algorithm as et al previously reported. Briefly, gene expression datasets were prepared using standard annotation files and data uploaded to the CIBERSORT web portal (http://cibersort.stanford.edu/), with the algorithm run using the default signature matrix at 1000 permutations. CIBERSORT derives a *P*‐value for the deconvolution for each sample using Monte Carlo sampling, providing a measure of confidence in the results.

CIBERSORT is a gene expression‐based deconvolution algorithm, it uses a set of barcode gene expression values (a “signature matrix” of 547 genes) for characterizing immune cell composition. In order to estimate the effect of variation in barcode genes, we used 644 cases from the TCGA cohort, where 100% of the genes are represented. We randomly deleted signature matrix genes in increments of 10% until 10% of genes remained to produce a graded representation of barcode genes matrix.

Immune cytolytic activity representing the geometric mean of GZMA and PRF1 is another in silico measure of immune infiltration, as described by Rooney et al^31^ Immune cytolytic activity for the TCGA and GEO datasets was determined by calculating the geometric mean of GZMK and PRF1.

### 2.3 Immunohistochemical detection of immune cell types

Tissue microarrays containing the specimens from 30 CRC patients who received curative surgery in the first affiliated hospital of Chongqing medical university (Chongqing, China) from April 2016 to September 2017 were constructed for immunohistochemistry. Specimens were all confirmed by pathological analysis as colorectal cancer. Immunohistochemistry was performed as described earlier,^32^, ^33^ using monoclonal antibodies against CD3 (SP7), CD8 (4B11), CD45RO (OPD4), CD57 (NK1), tryptase (AA1), CD1A (Ab‐5), granulocytes (BM‐2), PDPN (D2‐40), cytokeratin (AE1AE3), FOXP3 (ab20034; AbCam, Cambridge, United Kingdom) CD68 (PGM‐1), CD20 (L26; DAKO, Carpinteria, CA), IL3RA (IL3RA; ATLAS Antibodies, Stockholm, Sweden), CXCR5 and IL‐17 (H‐132; Santa Cruz Biotechnology, Santa Cruz, CA). Isotype‐matched mouse monoclonal antibodies were used as negative controls. Slides were analyzed using an image analysis workstation (Spot Browser, ALPHELYS). Polychromatic high‐resolution spot‐images (740 × 540 pixel, 1.181 μm/pixel resolution) were obtained (x200 fold magnification). The density was recorded as the number of positive cells per unit tissue surface area. For each duplicate, the mean density was used for statistical analysis.

### 2.4 Statistical analyses

Cases with a CIBERSORT *P*‐value of <0.05 were included in the main survival analysis. Median of the proportion of each cell type were computed for survival analysis and modeled as continuous variables in order to derive more easily interpretable hazard ratios (HRs). Associations between inferred proportions of immune cell types and survival were tested using Cox regression. Immune cell subsets significantly associated with outcome in unadjusted analyses were included in multivariable models. Multivariable analyses were adjusted for age, sex, local invasion, lymph node metastasis, distant metastasis, and TNM stage. The associations of immune cell infiltrate and corresponding disease‐free survival were analyzed by Kaplan‐Meier curves and evaluated using log‐rank test.

To assess the association between immune infiltration and response to chemotherapy, we identified and extracted patients who received chemotherapy after surgery from TCGA and GEO cohort if the relevant clinical information available and further investigated the influence of chemotherapy in different stratification.

To investigate whether distinct classes of immune cell infiltration are present in different tumors and whether these classes are associated with different clinical outcome, we conducted hierarchical clustering of immune cell proportions. Values were rescaled to lie between zero (for the smallest value observed) and one (for the greatest value observed) for each cell type to ensure comparability between rare (low overall proportion) and abundant (high overall proportion) cell types. Hierarchical clustering of these data by Ward's method was conducted across all samples. A combination of the Elbow method and the Gap statistic was used to explore the likely number of distinct clusters in the data. The associations between clusters and clinical outcome were tested using the methods described above.

All analyses were conducted using R version 3.3. All statistical tests performed were two‐sided, and the *P* values <0.05 were considered as statistical significance.

## 3 RESULTS

### 3.1 The performance of CIBERSORT for characterizing TIICs composition in CRC

**Figure 2:**
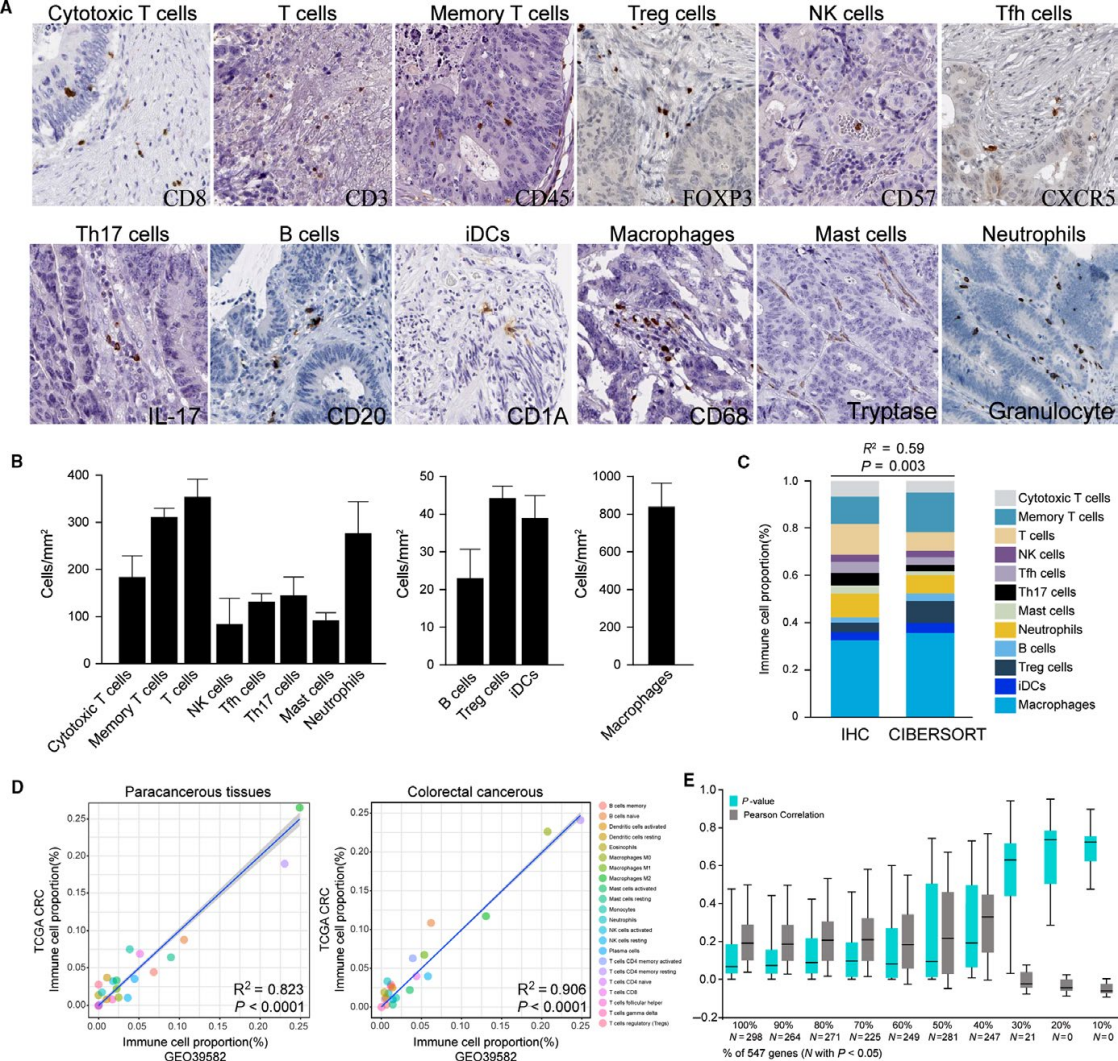
The performance of CIBERSORT for characterizing TIICs composition in CRC. A, Representative immunohistochemical images of infiltrated immune cells in CRC. T cells (quantified with marker CD3), cytotoxic T cells (CD8), memory T cells (CD45), Treg cells (FOXP3), activated T or NK cells (CD57), Tfh cells (CXCR5), Th17 cells (IL‐17), B cells (CD20), iDCs (CD1a), macrophages (CD68), mast cells (Tryptase), neutrophils (granulocyte) were stained and quantified by immunohistochemistry. B, The cell density of immune cell subpopulations. The density of the cells was recorded as the number of positive cells per mm^2^ surface area by use of a dedicated image‐analysis workstation (Spot Browser, Alphelys). To approximate ground truth proportions in CRC biopsies, levels were inferred by averaging TIICs counts from the tumor center and invasive margin of 30 patients. C, Relative TIICs proportions evaluated in CRC by CIBERSORT vs immunohistochemical analysis (IHC) on independent samples. CIBERSORT results are represented as mean TIICs proportions obtained from TCGA CRC cohort. D, Relative proportions of 22 TIICs subpopulation, as inferred by CIBERSORT, are compared between two independent datasets (TCGA CRC and GSE39582). E, Box plot of the distribution of CIBERSORT *P*‐value and average Pearson's correlation using datasets with progressively fewer (10% increments) barcode genes for 644 cases from the TCGA cohort

Although CIBERSORT coupled with LM22 allows for highly sensitive and specific discrimination of human leukocyte subsets, which had already applied in many previous studies.^22^, ^23^, ^24^ The realistic performance of CIBERSORT in CRC is not validated. To assess if the CIBERSORT could maintain its accuracy in CRC tissue, we applied an indirect compare between genomic and in suit immunohistochemistry analysis. Using tissue microarray, we examined the different TIICs subpopulations in CRC tissue of 30 patients (Figure 2A). All the TIICs subsets tested were found within the tumor at varying cell densities (Figure 2B). When compared against above immunohistochemistry experiment, the result of CIBERSORT based on analyzing TCGA CRC genomic data shown a high degree of consistent (*R* ^2 ^= 0.59, *P *=* *0.003; Figure 2C), which means CIBERSORT could accurately discriminate the proportions of TIICs subpopulation in CRC. Additionally, the relative proportions of 22 TIICs subpopulation, as inferred by CIBERSORT, are compared between two independent datasets (TCGA CRC and GSE39582) both containing colorectal tumor and adjacent normal specimens. Although above genomic profiles were obtained using different technologies and specimen sources, the proportions of TIICs subpopulation highly correlated and did not show any evident cohort bias (Figure 2D). Moreover, as shown in Figure 2D, most TIICs subpopulation present with significant discrepancy in relative fractions. These data combined with previous studies^21^ indicated that CIBERSORT was powerful enough to discriminate TIICs subpopulation in CRC.

CIBERSORT is a gene expression‐based deconvolution algorithm; it coupled with LM22, a defined “barcodes” with 547 gene expression signatures that distinguish 22 immune cell subpopulations (Table S1), and empirically defined global *P‐*value for characterizing immune cell composition. To evaluate the influence of *P‐*value and barcode genes on CIBERSORT performance, we next comparative analyzed the CIBERSORT outcome across 644 cases from the TCGA cohort where barcode genes were randomly removed in increments of 10%. As expected, the *P*‐value was highly sensitive to diminishing representation of the barcode genes (Figure 2E). However, of the samples with *248*, TIICs proportions remained relatively stable even if the barcode genes diminished from 100% to 40% (Figure S1). These findings imply that, while variation in barcode genes greatly influences the measurement accuracy of the TIICs subpopulations, this effect is not significant when analyses are limited to samples with *P *<* *0.05. Thus, unless otherwise specified, analyses in the current study were restricted to samples with *P *<* *0.05 and barcode genes >40%.

### 3.2 The landscape of immune infiltration in CRC

**Figure 3:**
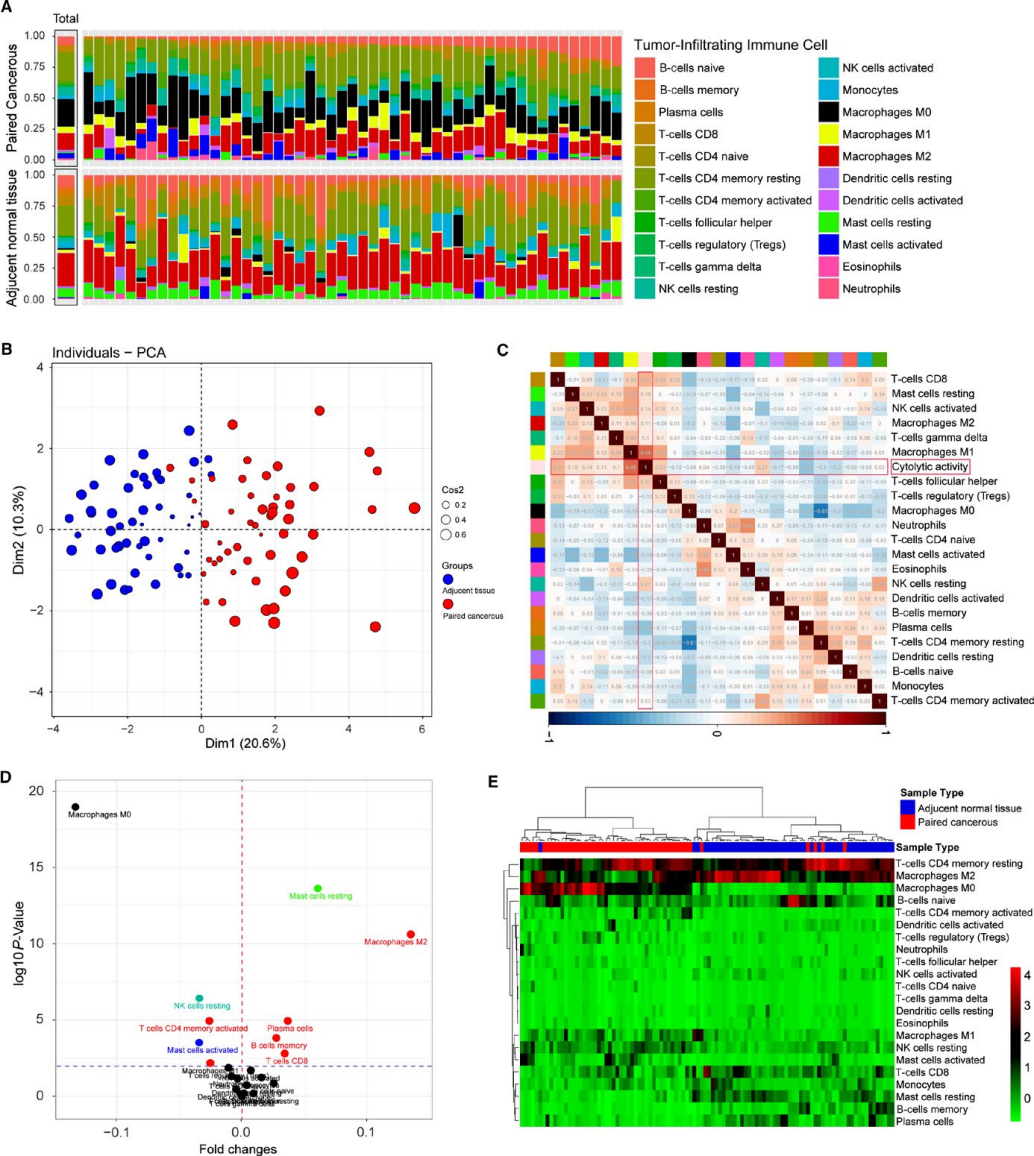
The landscape of immune infiltration in CRC. A, The difference of immune infiltration between paired cancer and paracancerous tissue. B, Principal components analysis performed on all paired CRC samples. The first two principal components which explain the most of the data variation are shown. C, Correlation matrix of all 22 immune cell proportions and immune cytolytic activity in the TCGA cohort. Variables have been ordered by average linkage clustering. D, Volcano Plot visualizing the differentially infiltrated immune cells. The red and blue points in plot represent the differentially subpopulations with statistical significance (*P* < 0.05). E, Heat map of the 22 immune cell proportions. The horizontal axis shows the clustering information of samples which were divided into two major clusters

Owing to technical limitation, the landscape of immune infiltration in CRC has not been entirely revealed, especially those low abundance cells subpopulation. Using CIBERSORT algorithm, we first investigated the difference of immune infiltration between paired cancer and paracancerous tissue in 22 subpopulations of immune cells. Figure 3A summarizes the results obtained from 32 CRC patients. Detailed results are provided in Table S2. Obviously, the proportions of immune cells in CRC varies significantly between both intra‐ and intergroup (Figure 3A). Thus, we speculated that variation in TIICs proportions might be an intrinsic feature which could characterize the individual differences. Indeed, the proportions of immune cells from 32 paired tissues displayed distinct group‐bias clustering and individual differences by PCA (Figure 3B), while the proportions of different TIICs subpopulations were weakly to moderately correlated (Figure 3C). Compared with paracancerous tissue, CRC tissue generally contained a higher proportion for M0, M1, NK cells resting, plasma cell, and T‐cells CD4 memory activated, whereas the mast cells resting and M2 fraction was relatively lower (Figure 3D, *P* < 0.05). Of note, above significantly changed TIICs subpopulation also contribute most to the distinct group‐bias clustering in PCA analysis (Figure S2). Moreover, as shown in Figure 3E, using unsupervised hierarchical clustering based on above‐identified cells subpopulation, the samples of tumor and normal were clearly separated into two discrete groups. Together, these results indicated that aberrant immune infiltration and its heterogeneous in CRC as a tightly regulated process might have important clinical meanings.

### 3.3 CIBERSORT *P*‐values reflect the overall proportion of immune cells

**Figure 4:**
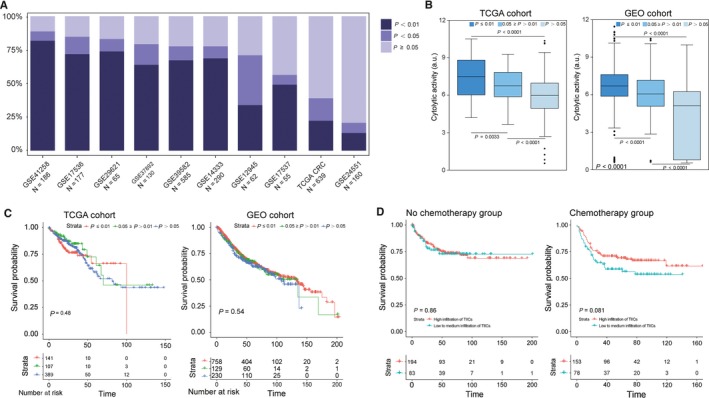
CIBERSORT *P*‐values reflect the overall proportion of immune cells. A, The proportion of samples with different *P*‐value threshold between studies. B, Box plots depicting the association between immune cytolytic activity and CIBERSORT *P*‐value, depicted *P*‐values are from Kruskal‐Wallis tests. C, Survival plots of groups defined by CIBERSORT *P*‐value separately by data source (TCGA, GEO), depicted *P*‐values are from log‐rank tests. D, Effect of overall immune infiltration on disease‐free survival in patients with records of chemotherapy or not. The survival of CRC patient subgroup with tumours containing low and high immune infiltration by CIBERSORT *P*‐value, depicted *P*‐values are from log‐rank tests

CIBERSORT algorithm only provides information about relative proportions among TIICs subpopulation, instead of actual value, which makes the results are not independent of each other. Of note, there were large differences in the proportions of samples with *P*‐value <0.05 between studies, spanning the whole range of 20.63% through 88.72% of samples (Figure 4A). We speculated that the *P*‐value derived by CIBERSORT would reflect the proportions of a sample that comprises immune cells vs nonimmune cells, where a greater proportion of immune cells would produce a corresponding smaller *P*‐value. To validate this speculation, we tested CIBERSORT *P*‐value against immune cytolytic activity, which was defined by Rooney et al as the geometric mean of GZMA and PRF1 expression, in the two largest datasets: GEO and TCGA. Strong ordinal relationship existed between different *P*‐value thresholds and cytolytic activity in both the GEO and TCGA datasets (Figure 4B). Besides, cytolytic activity was most strongly correlated with the proportion of CD8^+^ T cells (Pearson correlation = 0.31) and M1 macrophages (Pearson's correlation = 0.49) in the TCGA and GEO cohort at a CIBERSORT *P* < 0.05 (Figure 3C). Collectively, these results strongly indicate that the *P*‐value reflects the relative proportion of a sample composed of immune vs nonimmune cells.

In further exploring the association between *P*‐value thresholds and survival, we found patients with *P *<* *0.01, corresponding to a greater proportion of immune cells, were not associated with improved survival. Actually, as shown in Figure 4C, stratifying patients according to *P*‐value thresholds formed nonsignificant Kaplan‐Meier curves. Above results directly suggested that the prognostic effect of TIICs may mainly rely on the quantity of certain subsets rather than entirety.

Although there is still controversy, chemotherapy is universally recommended for CRC patients with stage II or III disease. Intriguingly, we noted that in those patients (TNM stage II or III) who were defined as high infiltration of immune cells (CIBERSORT *P *<* *0.01) had a favorable response to adjuvant chemotherapy. But for the same group of patients who have not received chemotherapy, high infiltration of immune cells could not improve the prognosis (Figure 4D). Collectively, these results revealed that CRC patients with increased infiltration of immune cells may benefit more from chemotherapy.

### 3.4 Identification of prognostic subsets of TIICs in CRC

**Figure 5:**
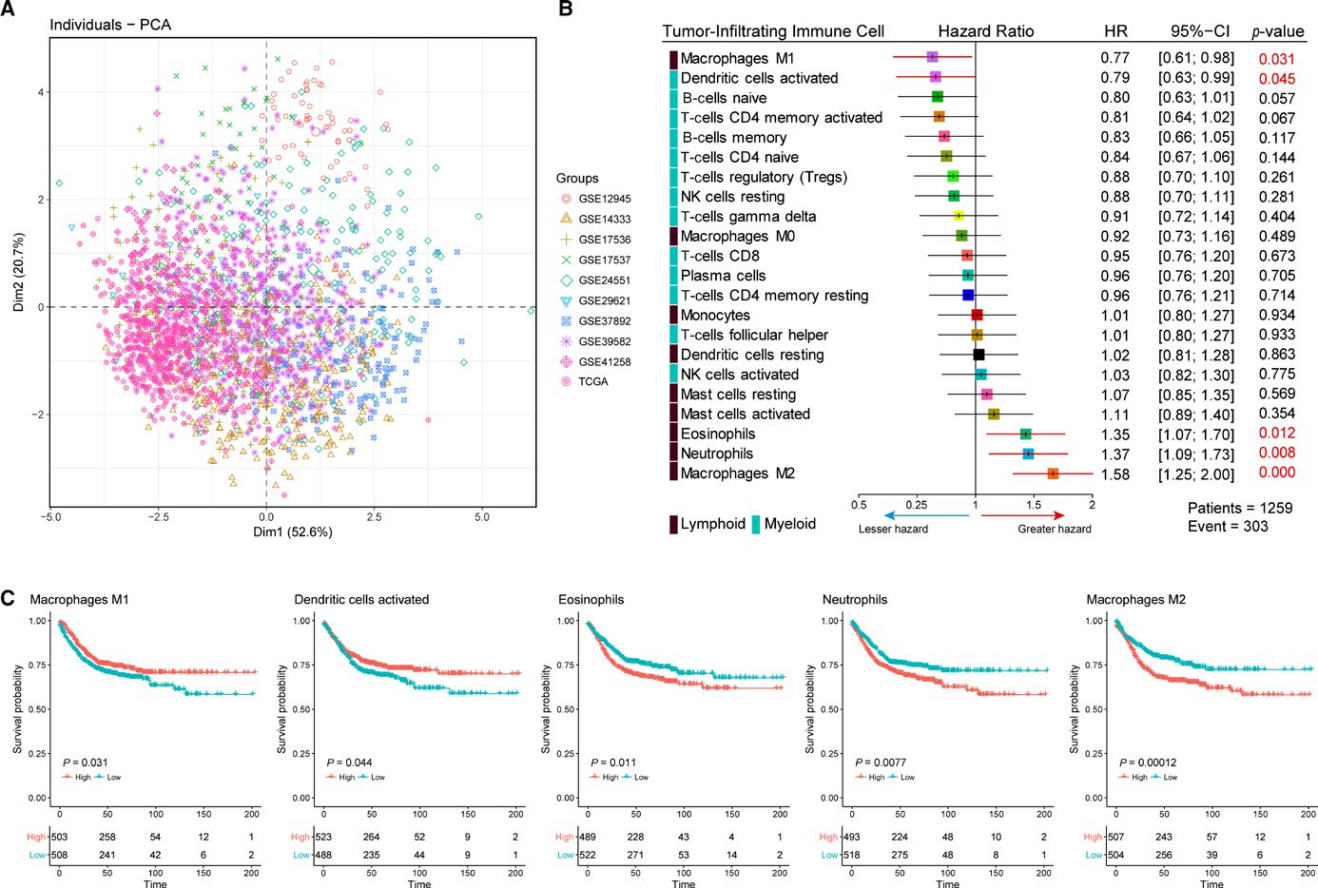
Identification of prognostic subsets of TIICs in CRC. A, Scatter plots depicting the variation of 22 TIICs subpopulations cross all sample from GEO and TCGA. The first two principal components which explain the most of the data variation are shown. Patients are labeled with different color according to the cohort to which they belong. B, Prognostic associations of TIICs subpopulation. Unadjusted HRs (boxes) and 95% confidence intervals (horizontal lines) limited to cases with CIBERSORT *P*‐value <0.05. Box size is inversely proportional to the width of the confidence interval. Number marked in red denote estimates with a *P*‐value <0.05. C, Survival plots of median of immune cell subsets. Depicted *P*‐values are from log‐rank tests.HR, hazard ratio

Although the genomic profiles were obtained using different technologies, owing to the fact that the proportion of TIICs subsets were obtained using uniform algorithm and they did not show any evident cohort‐bias clustering (Figure 5A). Thus, we combined the cohort form TCGA and GEO, and further investigated whether there was TIICs subpopulation statistically correlated with CRC‐related recurrence/progress by univariate Cox regression analysis. The unadjusted HRs and 95% confidence intervals for the median proportion of TIICs subsets is depicted in Figure 5B. Detailed results are provided in Table S3. Macrophages M1 (hazard ratio [HR] = 0.77, 95% CI = 0.61‐0.98; *P *=* *0.031) and dendritic activated cells (HR = 0.79, 95% CI = 0.62‐0.99; *P *=* *0.045) were significantly associated with improved outcome, whereas eosinophils (HR = 1.35, 95% CI = 1.07‐1.70; *P *=* *0.012), neutrophils (HR = 1.37, 95% CI = 1.08‐1.73; *P *=* *0.008), and macrophages M2 (HR = 1.58, 95% CI = 1.25‐2.01; *P < *0.001) were associated with poorer outcome. Kaplan‐Meier curve and log‐rank test for above‐identified TIICs subsets and the rest are shown in Figures 5C and S3, respectively.

We next assessed whether those selected TIICs subsets represent an independent indicator in CRC patients. Multivariable analyses adjusted for known prognostic factors revealed that, besides TNM stage, distant metastasis (M0/M1) and Local invasion (T3‐T4/T1‐T2) and lymph node metastasis (N0/N1‐N2), relative proportions of Macrophages M1 (HR = 0.58, 95% CI = 0.42‐0.82; *P *=* *0.002), neutrophils (HR = 1.41, 95% CI = 1.00‐1.98; *P *=* *0.049), and marophages M2 (HR = 1.34, 95% CI = 1.05‐1.897; *P *=* *0.042) was independent prognostic factors for DFS (Table S3), which means those TIICs subsets could add additional prognostic value to the current stage system.

### 3.5 Immune clusters associated with prognosis and molecular subtypes

**Figure 6:**
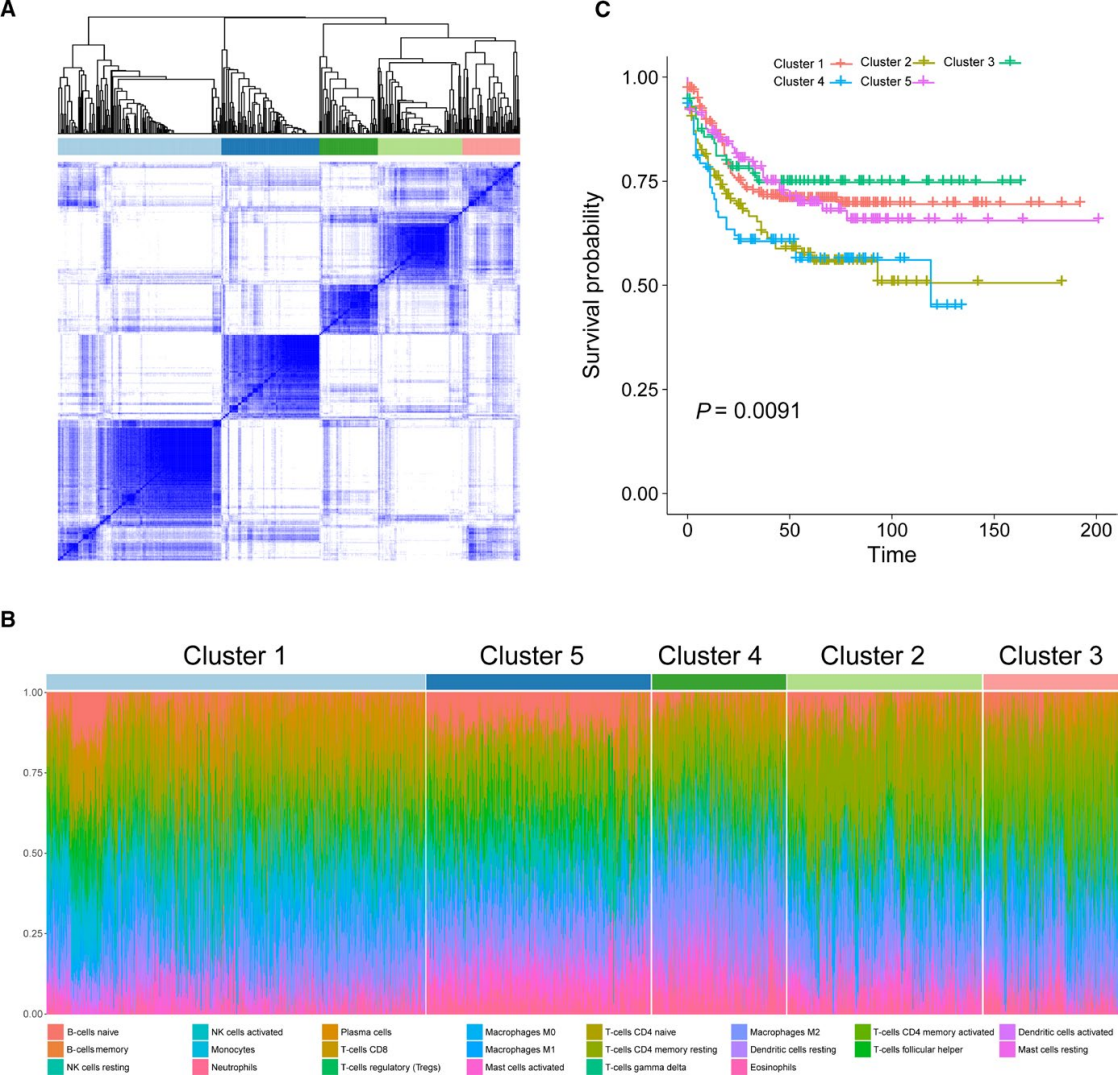
Immune clusters associated with prognosis and molecular subtypes. A, Consensus matrix heatmap defining five clusters of samples for which consensus values range from 0 (in white, samples never clustered together) to 1 (dark blue, samples always clustered together). B, Hierarchical clustering of all samples based on immune cell proportions. Stacked bar charts of samples ordered by cluster assignment. C, Kaplan‐Meier survival analysis of patients within different clusters. Depicted *P*‐values are from log‐rank tests

According to our findings, the variation of TIICs subsets change considerably at individual level and partly reflect the prognosis. We wonder whether distinct patterns of immune infiltration could be discerned based on the 22 TIICs subproportions by performing hierarchical clustering of all samples. The optimal number of clusters was determined by combining Elbow method and Gap statistic method, and the more balanced partition, as suggested, appeared to be for *k* = 5 (Figure S4). The consensus matrix heatmap revealed the identified five clusters, among which C1, C4, and C5 appeared as well individualized clusters, whereas there was more classification overlap between C2 and C3 (Figure 6A). The cell proportions of each clusters were shown in Figure 6B, and their distributions were depicted as box plots in Figure S5. Moreover, clusters were associated with distinct patterns of survival (Figure 6C). For example, cluster 4, defined by high levels of M2 macrophages, and cluster 2, defined by a relative high level of M2 macrophages and CD4 memory resting T cells, were both associated with poor outcome. In contrast, cluster 5, defined by moderate M1 macrophages and plasma cells and a high level of NK resting cells, was associated with improved outcome (Figure S5). More importantly, clusters were also significantly associated with previously defined molecular subtype (*P *<* *0.001; Figure S6). Collectively, these findings not only suggested that there is considerable variability in the nature of the immune infiltrate across CRC, which partly determined by the molecular characteristics of tumour, but also revealed that immune clusters could influence clinical outcome.

## 4 DISCUSSIONS

In addition to malignant neoplastic cells, cancer tissues also include immune cells, fibroblasts, endothelial cells, and an abundant collection of cytokines, chemokines, growth factors.^10^ Those components and their complicated interaction form the tumor‐related microenvironment which can exert inhibitory effects on even aggressive malignant cell, but during their progression, tumor cells may circumvent these inhibitory signals and instead exploit immune cells and others for their own benefits, resulting in growth, invasion, and metastasis.^34^, ^35^, ^36^ The predominant host cells recruited to and activated in the tumor microenvironment are various immune cells.^10^ It is well recognized that between immune cells and malignant cells in the tumor stroma, there is in fact a complex biological process which has significant prognostic relevance as the immune system has both tumor‐promoting and tumor‐inhibiting roles.^37^ In CRC, there is a marked infiltration of different types of immune cells, and the distribution, tissue localization, and cell types are significantly associated with progression and survival. For example, patients with stage III CRC whose tumors had low TIICs, the 5‐year OS and DFS rates were significantly lower as compared to the high TIICs group.^38^ In addition, high infiltration of TIICs in rectal cancer biopsies was associated with improved tumor response to preoperative radiochemotherapy and was significantly correlated with prolonged disease‐free and overall survival.^39^ These results providing evidence that immune cell infiltration represents not only a favorable prognostic factor but also could be predictive for the outcome of chemotherapies.

Despite growing studies shown that TIICs present with great promise in predicting the clinical outcome and treatment response at the individual level. One emerging question is whether certain subpopulation of TIICs plays a major role in influencing prognosis instead of overall infiltration. Actually, many previous studies demonstrated that certain TIICs subpopulation such as increased mature T cells, dendritic cells, and memory T‐cells infiltration are commonly related with favorable prognosis, while immune suppressive regulatory T cells are opposite.^15^, ^16^, ^17^ However, owing to the technical limitation, previous studies have been limited to a very narrow view of immune response. They evaluated TIICs by immunohistochemistry‐based analysis, which depends on a single surface marker to identify TIICs subpopulation. Obviously, this approach considerably less effective for discriminating closely related cell types and can be misleading as many marker proteins are expressed in different cell types. Thus, inconsistent results could usually be observed in clinical researches.

Advances in computational methods have reinvigorated the potential of large public repositories of genomic data collected over the past two decades. Surprisingly, integrating genomic profiles and the state‐of‐the‐art deconvolution algorithm, it is now possible to accurately resolve relative proportions of diverse TIICs subpopulation and overcome the defect of traditional immunohistochemistry‐based method.^40^, ^41^ Thus, in the current study, using a silicon analyses, known as CIBERSORT,^21^ to infer the proportions of 22 immune cell subsets from CRC transcriptomes, we have performed, to our knowledge, the most comprehensive analysis of the clinical impact of the immune response in CRC to date.

However, as an emerging technologies, CIBERSORT was only conducted in breast cancer,^24^ lung cancer,^22^ and artificial leukocyte signature matrix.^41^ The realistic performance of CIBERSORT in CRC is not validated. Thus, before our further analysis, we first applied an indirect compare between genomic and in suit immunohistochemistry analysis. We found that CIBERSORT was not only powerful enough to discriminate TIICs subtype in CRC, but also could remain consistency cross different genomic data resources. Those data were well in line with the results obtained by Gentles et al in comparing flow cytometry and immunohistochemistry experiment with CIBERSORT results.^42^ In addition, we also assessed the influence of *P‐*value and barcode genes on CIBERSORT performance. We found that the number of barcode genes greatly influences the accuracy of discriminating TIICs subpopulation, while this effect is not significant when analyses are limited to samples with *P *<* *0.05.

As an important statistical value, Raza Ali and his colleagues^24^ speculate that *P*‐value derived by CIBERSORT would reflect the proportion of a sample that comprises immune cells vs nonimmune cells, and finally validated their hypothesis. In the present study, we also obtained similar conclusions in CRC by using the same verification process. Recent studies demonstrated that the presence of TIICs was correlated with increased survival in the patients who had complete responses after chemotherapy.^2^, ^43^ In further exploring the association between overall proportion of immune cells (*P*‐value thresholds) and survival, we also found CRC patients with increased infiltration of immune cells may benefit more from chemotherapy.

Previously, it was thought that CRC was not an immunogenic cancer type, in contrast to melanoma or breast cancer.^44^ However, our unbiased method clearly confirmed that the immune infiltration is indeed involved in colorectal tumorigenesis. Using CIBERSORT, we directly compared the alteration of 22 subpopulations of immune cells between paired CRC and adjacent normal tissue for the first time, and found significant change occurred in both intra‐ and intergroup. More importantly, proportions of immune cells from 51 paired tissues displayed distinct group‐bias clustering and individual differences by PCA, which indicated that the variation of TIICs subtype as an intrinsic feature of CRC could characterize the individual differences and have important clinical meanings. Additionally, our data first revealed the detail of infiltration of 22 TIICs subsets in CRC that the proportions of macrophages account for more than 30%, in which 21% is M0, 13% is M2, whereas M1 only make up 5%. Moreover, CD4 memory resting T cells as a single TIICs subset occupied the biggest proportion (24%). As a subpopulation of T cells, CD4 memory resting T cells could further differentiation and been given a various function, including aid CD8^+^ T cells in tumor rejection, suppressing harmful immunological reactions to self‐ and foreign antigens and even blocking CD8^+^ T‐cell activation and NK cell killing.^45^, ^46^ Thus, it can be seen that CD4 memory resting T cells play a pivotal role in the development of CRC and its direction of differentiation could be a potential therapeutic target.

In univariate Cox regression analysis, we found that macrophages M1 (hazard ratio [HR] = 0.77, 95% CI = 0.61‐0.98; *P *=* *0.031) and dendritic activated cells (HR = 0.79, 95% CI = 0.62‐0.99; *P *=* *0.045) were significantly associated with improved outcome, whereas eosinophils (HR = 1.35, 95% CI = 1.07‐1.70; *P *=* *0.012), neutrophils (HR = 1.37, 95% CI = 1.08‐1.73; *P *=* *0.008) and macrophages M2 (HR = 1.58, 95% CI = 1.25‐2.01; *P < *0.001) were associated with poorer outcome. It is well known that the M1 (activated; anti‐tumoural) and M2 (alternatively activated; pro‐tumoral) phenotypes are associated with distinct immunoregulatory functions. Tumors are likely to change its macrophages subtype based on the microenvironment, which process represent a spectrum of functional states rather than truly distinct cell types,^22^ and our finding of an association between M1/M2 macrophages and improved/poorer outcome may reflect this gradation of function. We also found that the proportions of M1, and M2 macrophages defined several immune cell signatures in our clustering analysis, with prognostic implications. Moreover, neutrophils have been associated with angiogenesis and metastasis in animal models and increased neutrophil numbers are related to poor prognosis.^47^ The presence of high dendritic cells showed a trend toward an improved DFS.^16^ Our data came to confirm and extend the findings from previous studies mentioned above that it is the certain TIICs subpopulation instead of overall immune cell infiltration has the capacity to predict clinical outcomes. Interestingly, some TIICs subsets such as M0, mast cells resting, and NK cells resting differently infiltrated between tumor and adjacent normal tissue in CRC and the difference was statistically significant, which means above TIICs subsets play a role in colorectal tumorigenesis. However, we did not find association between those TIICs subsets and clinical outcomes. This may reflect the functional heterogeneity of TIICs subsets during the development of tumor.

Jérôme Galon et al defined the concept of cancer immune‐contexture, pioneered the immunoscore as a new method for routine clinical assessment of prognosis of patients with CRC.^48^, ^49^ According to the immunoscore, the most important cells associated with the prognosis are CD8^+^ cytotoxic T cells. However, in this study, the percentage of CD8^+^ T cells in entire TIICs were uncorrelated with DFS. The results in this study were obtained based on integrating gene expression profiles and computer algorithm. The radical difference in the methods between our and Jérôme Galon's study may the potential explanation for the discrepancy. Of note is that our results dose not negate the fact densities of CD8^+^ T cells allowed the stratification of patients into groups with different DFS rates.

Despite the significant results obtained in this present study, there were several shortcomings. In order to increase our sample size, we combined the clinical annotated genomic data from GEO and TCGA. Notwithstanding statistical methods has been conducted to eliminate cohort bias, heterogeneity in these data still impede the repeatability in some level. In addition, in our effort to obtain reliable estimates of association with clinical outcome, we collated diverse and heterogeneous studies and only remained the disease‐free survival, which with the highest integrality, and this process inevitably resulted in losing of some useful information.

In summary, our analysis of 22 immune cell subsets in CRC has revealed important associations with clinical outcome that have the potential to identify patients who could benefit from chemotherapy, as well as highlighting possible targets for new drugs. Coupling reliable deconvolution algorithms with large‐scale genomic data have the potential to further uncover the clinical and biological significance of the noncancer cells that comprise the tumor microenvironment in CRC.

## AVAILABILITY OF DATA AND MATERIALS

The datasets supporting the conclusions of this article are available in the in Gene Expression Omnibus (GEO) with the accession numbers listed as below: GSE12945, GSE14333, GSE17536, GSE17537, GSE24551, GSE29623, GSE37892, GSE39582, GSE42258. All of those studies previously were approved by their respective institutional review boards.

## CONFLICT OF INTEREST

The authors declare that they have no competing interests.
