# Supplementary material for: Profiles of immune infiltration in colorectal cancer and their clinical significant: A gene expression‐based study
Source: Cancer Med. 2018 Aug 16;7(9):4496–508. doi: 10.1002/cam4.1745 (PMC6144159; doi:10.1002/cam4.1745)
Supplement: Supplementary file 1 [file CAM4-7-4496-s001.doc]

**
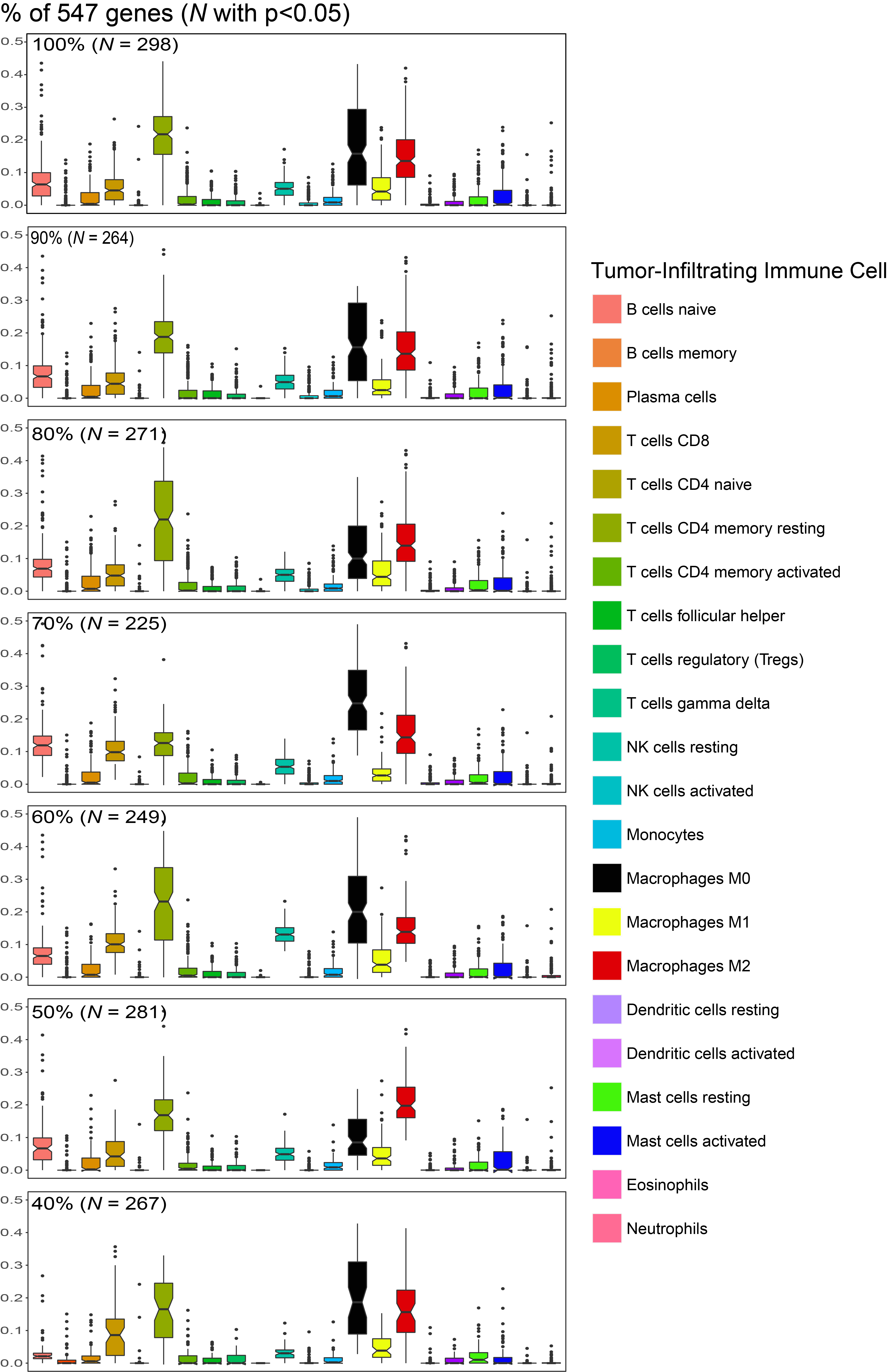
**

### **Fig. S1.** Distribution of 22 immune cell subsets for 644 cases from the TCGA study with decreasing representation of barcode genes.

**
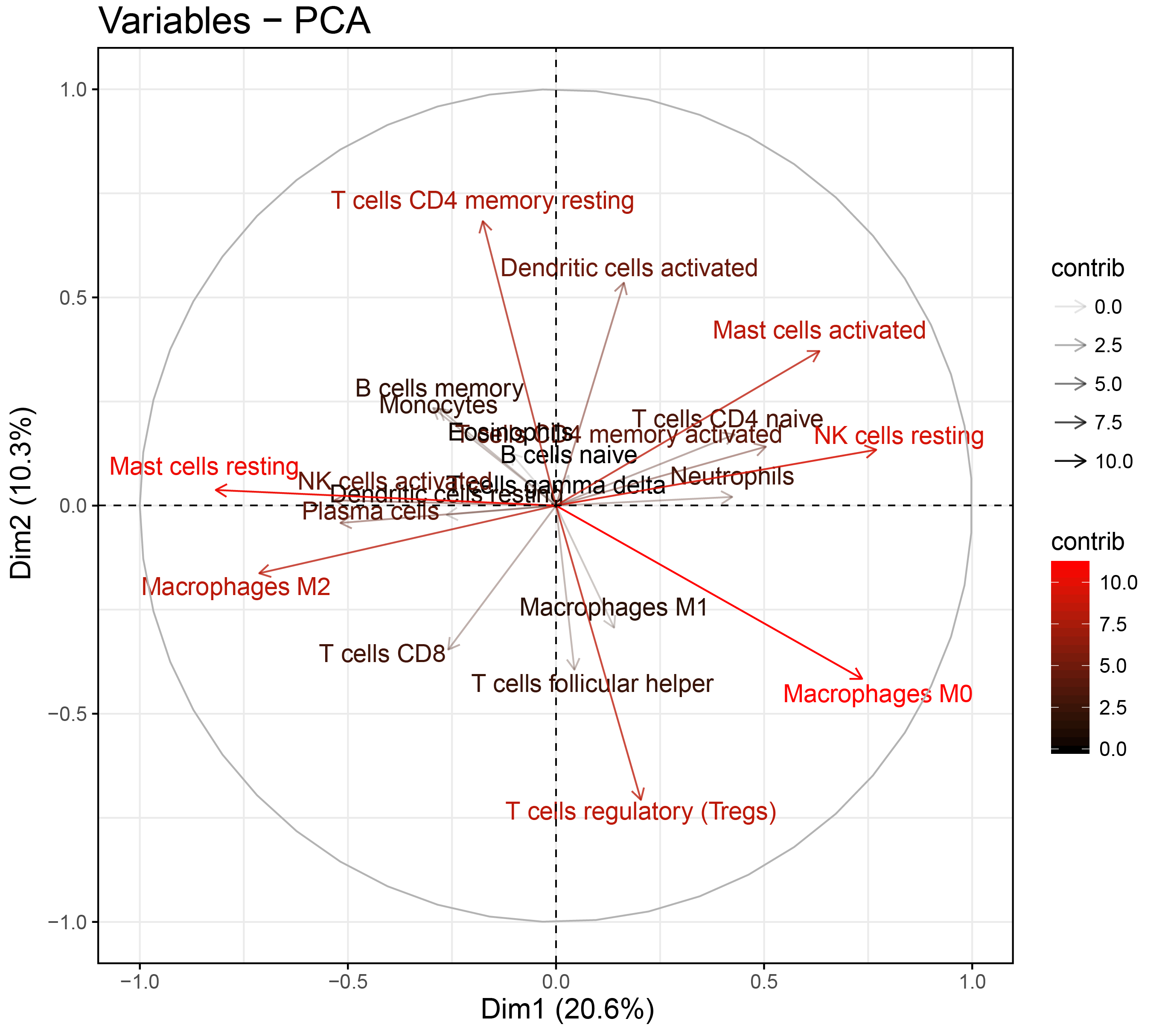
**

**Fig. S2. C**orrelation plots of 22 TIICs subpopulation.

**
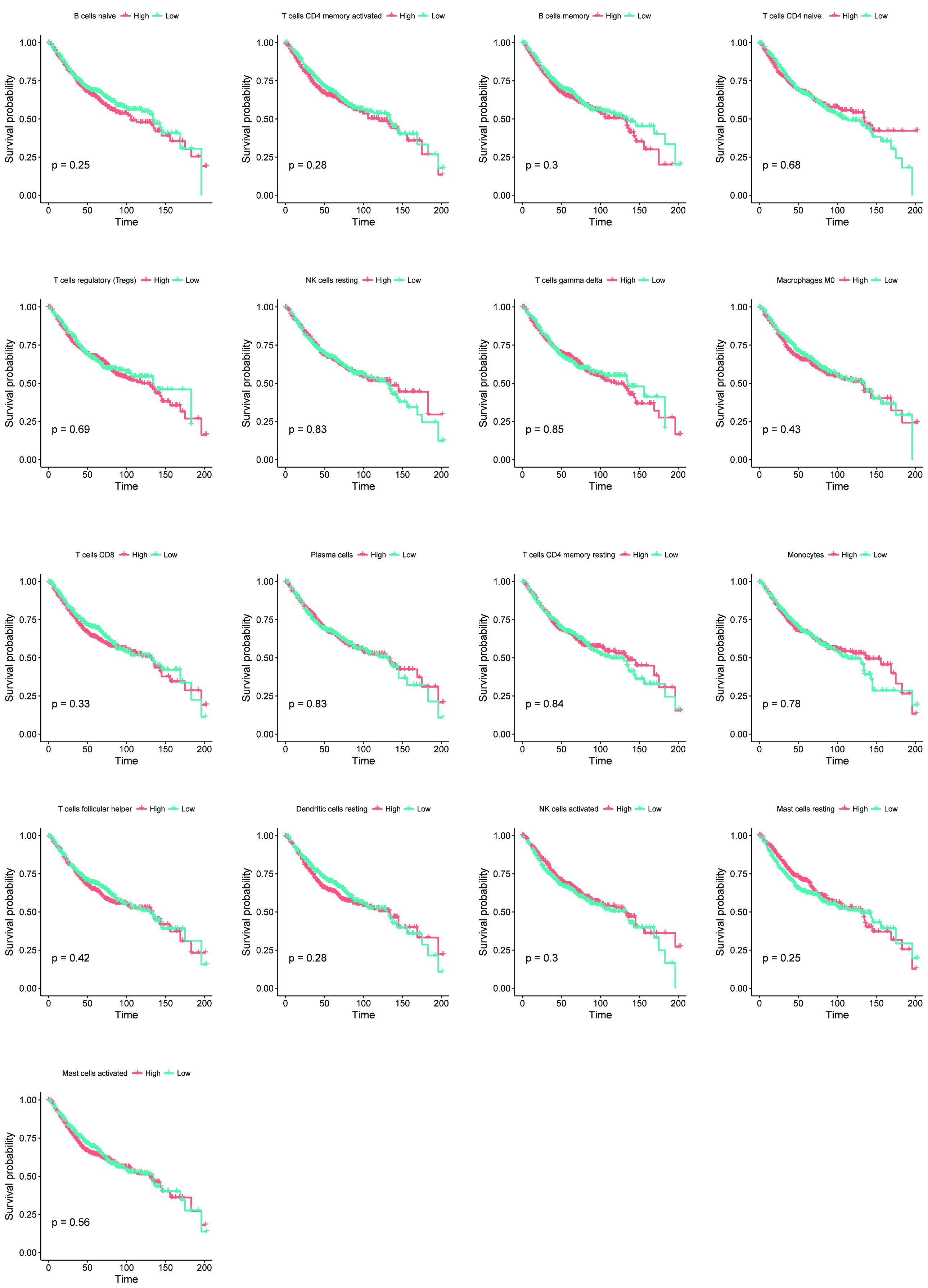
**

**Fig. S3.** The Kaplan-Meier survival curve of tumor-infiltrating immune cells in CRC.

**
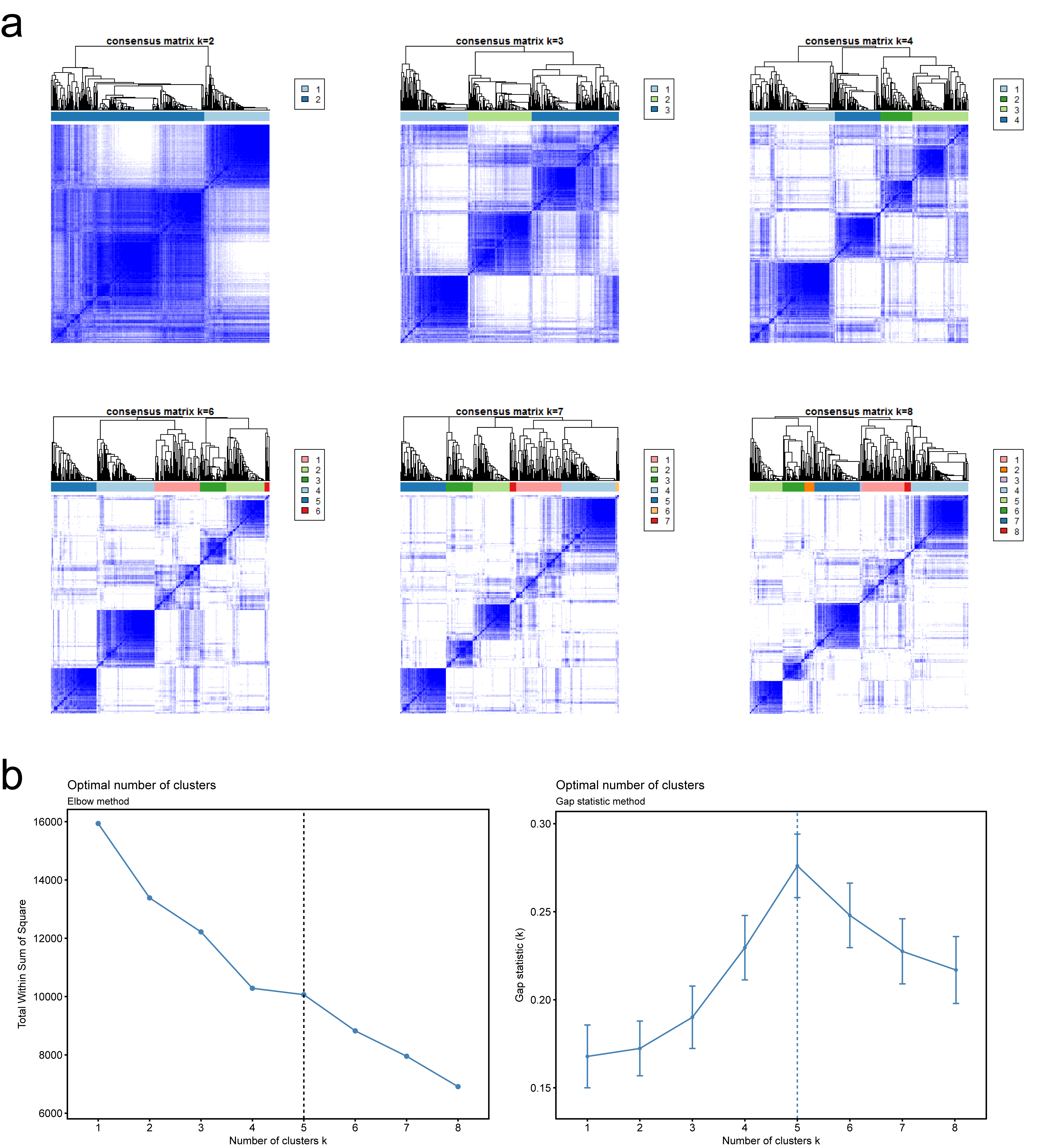
**

**Fig. S4. Selection of the number of clusters.** (a) consensus matrix for different numbers of clusters (*k* = 2 to 8). (b) Elbow and Gap statistic analysis for each tested number of clusters.


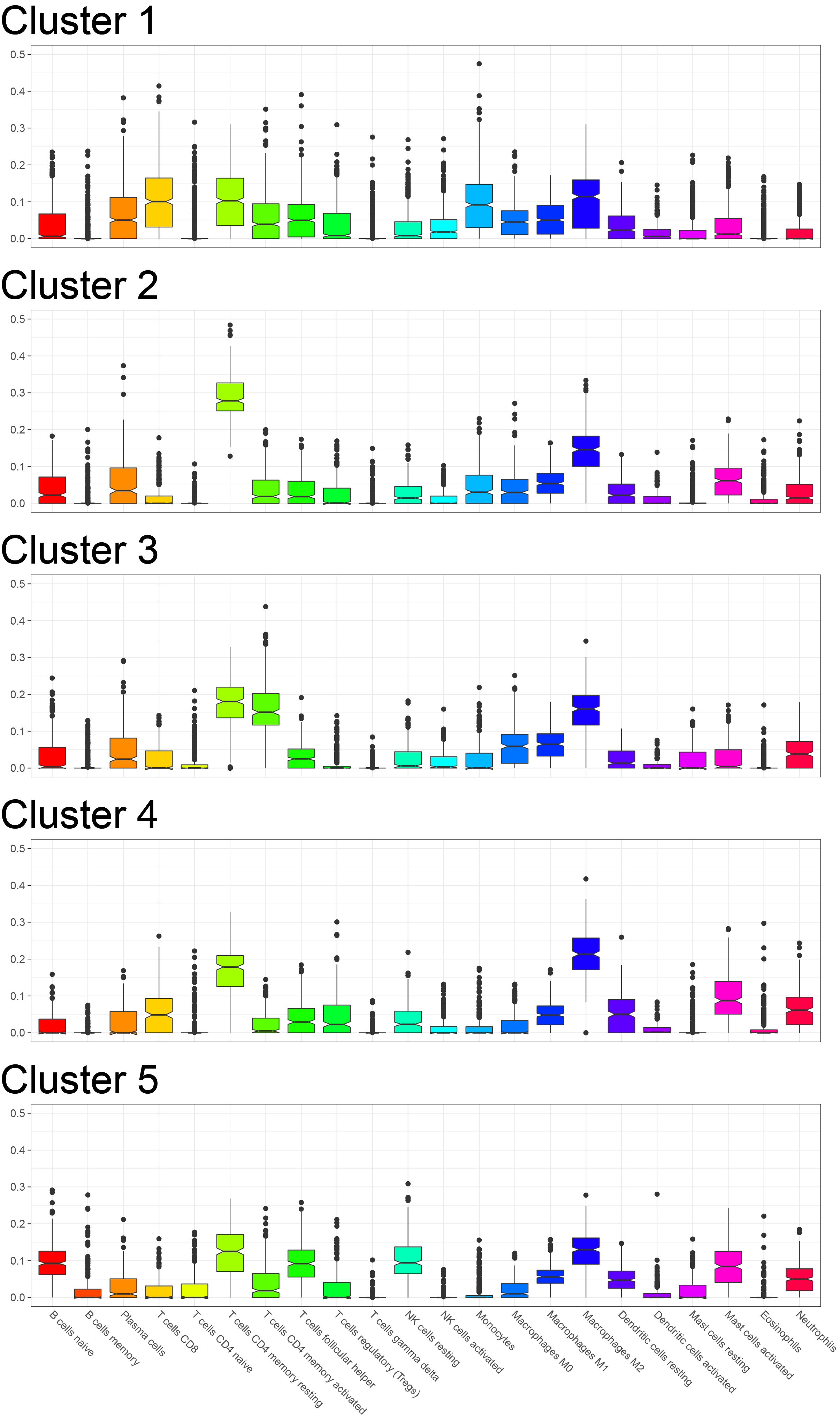


**Fig. S5.** The box plots of cell distributions of each clusters.

**
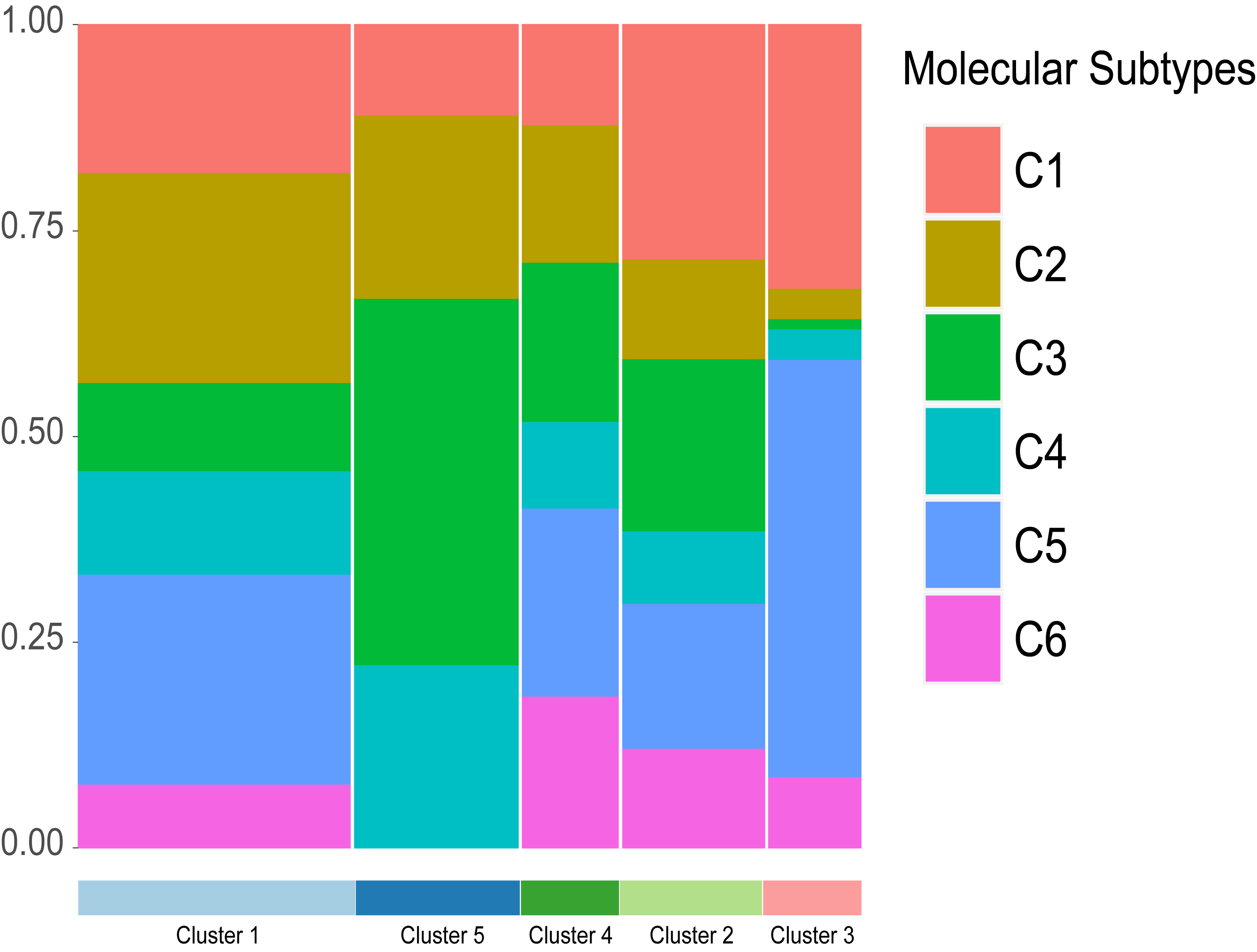
**

**Fig. S6.** Spine plots of the relationship between immune cluster and molecular subtype. *P-*values are from Kruskal-Wallis tests.
